# Individual Brain Charting dataset extension, second release of high-resolution fMRI data for cognitive mapping

**DOI:** 10.1038/s41597-020-00670-4

**Published:** 2020-10-16

**Authors:** Ana Luísa Pinho, Alexis Amadon, Baptiste Gauthier, Nicolas Clairis, André Knops, Sarah Genon, Elvis Dohmatob, Juan Jesús Torre, Chantal Ginisty, Séverine Becuwe-Desmidt, Séverine Roger, Yann Lecomte, Valérie Berland, Laurence Laurier, Véronique Joly-Testault, Gaëlle Médiouni-Cloarec, Christine Doublé, Bernadette Martins, Eric Salmon, Manuela Piazza, David Melcher, Mathias Pessiglione, Virginie van Wassenhove, Evelyn Eger, Gaël Varoquaux, Stanislas Dehaene, Lucie Hertz-Pannier, Bertrand Thirion

**Affiliations:** 1 Université Paris-Saclay, Inria, CEA, Palaiseau, 91120 France; 2 Université Paris-Saclay, CEA, CNRS, BAOBAB, NeuroSpin, 91191 Gif-sur-Yvette, France; 3 Cognitive Neuroimaging Unit, INSERM, CEA, Université Paris-Saclay, NeuroSpin center, 91191 Gif/Yvette, France; 4 Laboratory of Cognitive Neuroscience, Brain Mind Institute, School of Life Sciences and Center for Neuroprosthetics, Swiss Federal Institute of Technology (EPFL), Campus Biotech, Geneva, Switzerland; 5 Motivation, Brain and Behavior (MBB) team, Institut du Cerveau (ICM), Inserm UMRS 1127, CNS UMR 7225, Sorbonne Université, Paris, France; 6 Center for Mind/Brain Sciences, University of Trento, I-38068 Rovereto, Italy; 7 LaPsyDÉ, UMR CNRS 8240, Université de Paris, Paris, France; 8 GIGA-CRC In vivo Imaging, University of Liège, Liège, Belgium; 9 Institute of Neuroscience and Medicine, Brain & Behaviour (INM-7) Research Centre Jülich, Jülich, Germany; 10 Criteo AI Lab, Paris, France; 11 Université Paris-Saclay, CEA, UNIACT, NeuroSpin, 91191 Gif-sur-Yvette, France; 12 Collège de France, Université Paris-Sciences-Lettres, Paris, France; 13 UMR 1141, NeuroDiderot, Université de Paris, Paris, France

**Keywords:** Brain imaging, Cognitive neuroscience, Human behaviour, Functional magnetic resonance imaging

## Abstract

We present an extension of the *Individual Brain Charting* dataset –a high spatial-resolution, multi-task, functional Magnetic Resonance Imaging dataset, intended to support the investigation on the functional principles governing cognition in the human brain. The concomitant data acquisition from the same 12 participants, in the same environment, allows to obtain in the long run finer cognitive topographies, free from inter-subject and inter-site variability. This second release provides more data from psychological domains present in the first release, and also yields data featuring new ones. It includes tasks on e.g. mental time travel, reward, theory-of-mind, pain, numerosity, self-reference effect and speech recognition. In total, 13 tasks with 86 contrasts were added to the dataset and 63 new components were included in the cognitive description of the ensuing contrasts. As the dataset becomes larger, the collection of the corresponding topographies becomes more comprehensive, leading to better brain-atlasing frameworks. This dataset is an open-access facility; raw data and derivatives are publicly available in neuroimaging repositories.

## Abstract (summary)

**Table:** 

Measurement(s)	functional brain measurement • regional part of brain • brain measurement • cognition
Technology Type(s)	functional magnetic resonance imaging
Factor Type(s)	type of task
Sample Characteristic - Organism	Homo sapiens

Machine-accessible metadata file describing the reported data: 10.6084/m9.figshare.12958181

## Background & Summary

Understanding the fundamental principles that govern human cognition requires mapping the brain in terms of functional segregation of specialized regions. This is achieved by measuring local differences of brain activation related to behavior. *Functional Magnetic Resonance Imaging* (fMRI) has been used for this purpose as an attempt to better understand the neural correlates underlying cognition. However, while there is a rich literature concerning performance of isolated tasks, little is still known about the overall functional organization of the brain.

Meta- and mega-analyses constitute active efforts at providing accumulated knowledge on brain systems, wherein data from different studies are pooled to map regions consistently linked to mental functions^1–9^ Because data are impacted by both intra- and inter-subject plus inter-site variability, these approaches still limit the exact demarcation of functional territories and, consequently, formal generalizations about brain mechanisms. Several large-scale brain-imaging datasets are suitable for atlasing, wherein differences can be mitigated across subjects and protocols together with standardized data-processing routines. Yet, as they have different scopes, not all requirements are met for cognitive mapping. For instance, the *Human Connectome Project* (HCP)^10,11^ and *CONNECT/Archi*^12,13^ datasets provide large subject samples as they are focused in population analysis across different modalities; task-fMRI data combine here 24 and 28 conditions, respectively, which is scarce for functional atlasing. Another example is the *studyforrest* dataset^14–17^, that includes a variety of task data on complex auditory and visual information, but restricted to naturalistic stimuli. Additionally, one shall note that within-subject variability reduces task-fMRI replicability; thus, more data per subject can in fact facilitate reliability of group-level results^18^.

To obtain as many cognitive signatures as possible and simultaneously achieve a wide brain coverage at a fine scale, extensive functional mapping of individual brains over different psychological domains is necessary. Within this context, the *Individual Brain Charting* (IBC) project pertains to the development of a 1.5mm-resolution, task-fMRI dataset acquired in a fixed environment, on a permanent cohort of 12 participants. Data collection from a broad range of tasks, at high spatial resolution, yields a sharp characterization of the neurocognitive components common to the different tasks. This extension corresponds to the second release of the IBC dataset, meant to increase the number of psychological domains of the first one^19^. It both aims at a consistent mapping of elementary spatial components, extracted from all tasks, and a fine characterization of the individual architecture underlying this topographic information.

The first release encompassed a sample of modules ranging from perception to higher-level cognition, e.g. retinotopy, calculation, language and social reasoning^10,12,20^. The second release refers to tasks predominantly focused on higher-level functions, like mental time travel, reward, theory-of-mind, self-reference effect and speech recognition. Nonetheless, a subset dedicated to lower-level processes is also included, covering pain, action perception and numerosity. These tasks are intended to complement those from the first release, such that a considerable cognitive overlap is attained, while new components are introduced. For instance, components concerning social cognition, already found in *ARCHI Standard*, *ARCHI Social* and *HCP Social* tasks from the previous release, are now present in tasks about theory-of-mind and self-reference effect. Likewise, components on incentive salience, already tackled in the *HCP Gambling* task, are now included in a task battery addressing positive-incentive value. Yet also, a battery on mental time travel brings in new modules pertaining to time orientation and cardinal-direction judgment. Data from both releases are organized in 25 tasks –most of them reproduced from other studies– and they amount for 205 contrasts described on the basis of 110 cognitive atoms, extracted from the *Cognitive Atlas*^21^.

Here, we give an account –focused on the second release– of the experimental procedures and the dataset organization and show that raw task-fMRI data and their derivatives represent functional activity in direct response to behavior. Data collection is ongoing and more releases are planned for the next years. Despite being a long-term project, IBC is not dedicated to longitudinal surveys; acquisitions of the same tasks will not be conducted systematically.

The IBC dataset is an open-access facility devoted to providing high-resolution, functional maps of individual brains as basis to support investigations in human cognition.

## Methods

To avoid ambiguity with MRI-related terms used throughout this manuscript, definitions of such terms follow the *Brain-Imaging-Data-Structure* (BIDS) Specification version 1.2.1^22^.

Complementary information about dataset organization and MRI-acquisition protocols can be found in the IBC documentation available online: https://project.inria.fr/IBC/data/

### Participants

The present release of the IBC dataset consists of brain fMRI data from eleven individuals (one female), acquired between April 2017 and July 2019. The two differences from the cohort of the first release are: *(1)* the replacement of participant 2 (sub-02) by participant 15 (sub-15); and *(2)* the absence of data from participant 8 (sub-08). Regarding the latter, data will be acquired in the future and included in one of the upcoming releases.

Age, sex and handedness of this group of participants is given on Table 1. Handedness was determined with the Edinburgh Handedness Inventory^23^.

**Table 1:** Demographic data of the participants.

Subject ID	Year of recruitment	Age	Sex	Handedness score
sub-01	2015	39.5	M	0.3
sub-04	2015	26.9	M	0.8
sub-05	2015	27.4	M	0.6
sub-06	2015	33.1	M	0.7
sub-07	2015	38.8	M	1
sub-09	2015	38.5	F	1
sub-11	2016	35.8	M	1
sub-12	2016	40.8	M	1
sub-13	2016	28.2	M	0.6
sub-14	2016	28.3	M	0.7
sub-15	2017	30.3	M	0.9

Age stands for the participants’ age upon recruitment. All acquisitions of the present release took place between April 2017 and July 2019.

All experimental procedures were approved by a regional ethical committee for medical protocols in Île-de-France (“Comité de Protection des Personnes” - no. 14-031) and a committee to ensure compliance with data-protection rules (“Commission Nationale de l’Informatique et des Libertés” - DR-2016-033). They were undertaken with the informed written consent of each participant according to the Helsinki declaration and the French public health regulation. For more information, consult^19^.

## Materials

### Stimulation

For all tasks (see Section “Experimental Paradigms” for details), the stimuli were delivered through custom-made scripts that ensure a fully automated environment and computer-controlled collection of the behavioral data. Two software tools were used for the development of such protocols: *(1)* Expyriment (versions 0.7.0 and 0.9.0, Python 2.7); and *(2)* Psychophysics Toolbox Version 3 for GNU Octave version 4.2.1. The visual and auditory stimuli presented in the *Theory-of-Mind and Pain Matrices* battery as well as in the *Bang* task (see respectively Sections “Theory-of-Mind and Pain Matrices task battery” and task “Bang task” for details) were translated into French. The corresponding material is publicly available, as described in Section “Code Availability”.

### MRI Equipment

The fMRI data were acquired using an MRI scanner Siemens 3 T Magnetom Prisma^fit^ along with a Siemens Head/Neck 64-channel coil. Behavioral responses were obtained with two MR-compatible, optic-fiber response devices that were interchangeably used according to the type of task employed: *(1)* a five-button ergonomic pad (Current Designs, Package 932 with Pyka HHSC-1 × 5-N4); and *(2)* a pair of in-house custom-made sticks featuring one-top button. MR-Confon package was used as audio system in the MRI environment.

All sessions were conducted at the NeuroSpin platform of the CEA Research Institute, Saclay, France.

## Experimental Procedure

Upon arrival to the research institute, participants were instructed about the execution and timing of the tasks referring to the upcoming session.

All sessions were composed of several runs dedicated to one or a group of tasks as described in Section “Experimental Paradigms”. The structure of the sessions according to the MRI modality employed at every run is detailed in Table 2. Specifications about imaging parameters of the referred modalities as well as procedures undertaken toward recruitment of participant 15 plus handling and training of all participants are described in^19^. As a side note, data pertaining to tasks of the first release were also acquired for participant 15.

**Table 2:** Plan of the MRI-data acquisitions for the sessions pertaining the second release of the IBC dataset.

Session	Modality	Task *	Duration** (min:sec)	Repetitions
MTT1	2D Spin-Echo	—	00:31	PA(×2) + AP(×2)
	BOLD fMRI	MTT WE^†^	13:08	PA(×2) + AP
MTT2	2D Spin-Echo	—	00:31	PA(×2) + AP(×2)
	BOLD fMRI	MTT SN^‡^	13:08	PA(×2) + AP
Preference	2D Spin-Echo	—	00:31	PA(×2) + AP(×2)
	BOLD fMRI	Food	08:16	PA + AP
	BOLD fMRI	Painting	08:16	PA + AP
	BOLD fMRI	Face	08:16	PA + AP
	BOLD fMRI	House	08:16	PA + AP
TOM	2D Spin-Echo	—	00:31	PA(×2) + AP(×2)
	BOLD fMRI	TOM localizer	06:12	PA + AP
	BOLD fMRI	Emotional Pain localizer	05:12	PA + AP
	BOLD fMRI	Pain Movie localizer	05:56	PA + AP
Enumeration	2D Spin-Echo	—	00:31	PA(×2) + AP(×2)
	BOLD fMRI	VSTM	08:40	PA(×2) + AP(×2)
	BOLD fMRI	Enumeration	16:20	PA + AP
Self	2D Spin-Echo	—	00:31	PA(×2) + AP(×2)
	BOLD fMRI	Self 1–2	12:00	PA(×2)
	BOLD fMRI	Self 3	12:00	AP
	BOLD fMRI	Self 4${\rm{\S }}$	15:58	AP
	BOLD fMRI	Bang	08:06	PA

A BOLD-fMRI run refers to the acquisition of fMRI data on one single task. At least, there were two BOLD runs, corresponding to PA- and AP- phase-encoding directions for each task during a session. The 2D Spin-Echo PA/AP maps were always acquired before the runs dedicated to the collection of BOLD-fMRI data and repeated afterwards.

*Full descriptions of task siglas are provided in Section “Experimental Paradigms”.

**For BOLD fMRI sequences, the durations presented here account only for the period of the actual acquisition. The full duration of each run also included ∼45 s of calibration scans, always performed at their beginning.

^†^Mental Time Travel task featuring “West-East island” stimuli.

^‡^Mental Time Travel task featuring “South-North island” stimuli.

^§^The run “Self 4” relates to a longer version of the others runs of the same task. Thus, “Self 4” contains four pairs of *Encoding* + *Recognition* phases, whereas the remaining runs contain only three pairs.

## Experimental Paradigms

Tasks were aggregated in different sessions according to their original studies^24–33^. Most of the paradigms are composed by trials usually separated by the display of a fixation cross. All trials within each task were randomized in order to avoid the extensively consecutive repetition of trials containing conditions of the same kind. For some tasks, trials were in fact pseudo-randomized by following specific criteria relative to the experimental design of those tasks.

The following sections are thus dedicated to a full description of the set of paradigms employed for each task, including description of the experimental conditions, temporal organization of the trials and their (pseudo-)randomization. Moreover, Table 3 provides an overview of the tasks, which includes a short description and motivation of their inclusion in terms of psychological domains covered. Ideally and as mentioned in Section “Background and Summary”, the main purpose of each release is to provide the dataset with a greater variety of cognitive modules from as many new psychological domains as possible, at the same time that a better coverage with the already existing ones is also attained.

**Table 3:** Overview of the tasks featuring the second release of the IBC dataset.

Tasks	Description	Psychological Domains covered	References
MTT battery	Assess the mental time and space shifts involved in the allocentric mapping of fictional events described in terms of audio narratives.	*Existing*: auditory cognition, spatial cognition memory *New*: temporal cognition (e.g. time orientation) spatial cognition (e.g. cardinal orientation).	^24–26^
Preference battery	Assess decision-making associated with the positive-incentive value and level of confidence in the evaluation of visual constructs.	*Existing*: incentive salience, visual cognition perception *New*: confidence, food-cue responsiveness.	^27^
TOM battery	Assess theory-of-mind and pain-matrix networks.	*Existing*: language, social cognition, theory-of-mind *New*: pain.	^28–30^
VSTM + Enumeration	Assess numerosity with and without encoding of object features.	*Existing*: numerical cognition, visual cognition *New*: numerical cognition (e.g. numerosity).	^31^
Self	Assess the *Self-Reference Effect.*	*Existing*: recognition.	^32^
		*New*: Self (e.g. self-reference effect, episodic memory).	
Bang	Assess speech comprehension during movie watching.	*Existing*: language *New*: perception (e.g. action perception) auditory cognition (e.g. auditory-scene analysis).	^33^

The list contains a short description of every task along with a brief summary of the psychological domains addressed by its experimental conditions. The bibliographic references pertaining to their original studies are also provided in the right most column.

All material used for stimulus presentation have been made publicly available (see Section “Code Availability”), together with video annotations of the corresponding protocols. Video annotations refer to video records of complete runs that are meant to be consulted for a better comprehension of the task paradigms. For each subject, the paradigm-descriptors’ files describing the occurrence of the events are part of the dataset, following BIDS Specification.

### *Mental time travel* (MTT) task battery

The *Mental Time Travel* (MTT) task battery was developed following previous studies conducted at the NeuroSpin platform on chronosthesia and mental space navigation^24–26^. In these studies, participants judged the ordinality of real historical events in time and space by mentally project oneself, i.e. through egocentric mapping. In contrast, the present task was intended to assess the neural correlates underlying both mental time and space judgment involved in allocentric mapping implemented in narratives. To this end, and in order to remove confounds associated with prior subject-specific mental representations linked to the historical events, fictional scenarios were created with fabricated stories and characters.

Concretely, this battery is composed of two tasks –*MTT WE* and *MTT SN*– that were employed, each of them, in two different sessions. The stimuli of each task referred to a different island plotting different stories and characters. There were two stories per island and they were created based on a two-dimensional mesh of nodes. Each node corresponded to a specific action. The stories of each island evolved both in time and in one single cardinal direction. The cardinal directions, cued in the task, differed between sessions. Thus, space judgment was performed according to the cardinal directions *West-East* and *South-North* for tasks MTT WE and MTT SN, respectively. In addition, the stories of each island evolved spatially in opposite ways. For instance, the two stories plotted in the West-East island evolved across time from west to east and east to west, respectively.

Prior to each session, participants were to learn the story of the corresponding session. To prevent any retrieval of graphical memories referring to the schematic representation of the stories, they were presented as audio narratives. Additionally, the participants were also instructed to learn the stories chronographically, i.e. as they were progressively referred to in the narrative, and to refrain from doing (visual) notes, which could be encoded as mental judgments.

The task was organized as a block-design paradigm, composed of trials with three conditions of audio stimuli: *(1) Reference*, statement of an action in the story to serve as reference for the time or space judgment in the same trial; *(2) Cue*, question concerning the type of mental judgment to be performed in the same trial, i.e. *“Before or After?”* for the time judgment or *“West or East?”* and *“South or North?”* for the space judgment in the first and second sessions, respectively; and *(3) Event*, statement of an action to be judged with respect to the Reference and according to the Cue.

Every trial started with an audio presentation of the Reference followed by silence, with a duration of two and four seconds, respectively. The audio presentation of the Cue came next, followed by a silence period; they had respectively a duration of two and four seconds. Afterwards, a series of four Events were presented for two seconds each; all of them were interspersed by a *Response* condition of three seconds. Every trial ended with a silent period of seven seconds, thus lasting thirty nine seconds in total.

A black fixation cross was permanently displayed on the screen across conditions and the participants were instructed to never close their eyes. At the very end of each trial, the cross turned to red during half of a second in order to signal the beginning of the next trial; such cue facilitated the identification of the next audio stimulus as the upcoming Reference to be judged.

During the Response period, the participants had to press one of the two possible buttons, placed in their respective left and right hand. If the Cue presented in the given trial hinted at time judgment, the participants were to judge whether the previous Event occurred before the Reference, by pressing the button of the left hand, or after the Reference, by pressing the button of the right hand. If the Cue concerned with space judgment, the participants were to judge, in the same way, whether the Event occurred west or east of the Reference in the first session and south or north of the Reference in the second session.

One session of data collection comprised three runs; each of them included twenty trials. Half of the trials for a given run were about time navigation and the other half, space navigation. Five different references were shared by both types of navigation and, thus, there were two trials with the same reference for each type of navigation. Within trials, half of the Events related to past or western/southern actions and the other half to future or eastern/northen actions with respect to the Reference.

The order of the trials was shuffled within runs, only to ensure that each run would feature a unique sequence of trials according to type of reference (both in time and space) and cue. No pseudo-randomization criterion was imposed as the trials’ characterization was already very rich. Since there were only two types of answers, we also randomized events according to their correct answer within each trial. The same randomized sequence for each run was employed for all participants. The code of this randomization is provided together with the protocol of the task in a public repository on GitHub (see Section “Code Availability”). Note that the randomized sequence of trials for all runs is pre-determined and, thus, provided as inputs to the protocol for a specific session.

For sake of clarity, Online-only Table 1 contains a full description of all conditions employed in the experimental design of this task.

### Preference task battery

The *Preference* task battery was adapted from the *Pleasantness Rating task* (Study 1a) described in^27^, in order to capture the neural correlates underlying decision-making for potentially rewarding outcomes (*aka* “positive-incentive value”) as well as the corresponding level of confidence.

The whole task battery is composed of four tasks, each of them pertaining to the presentation of items of a certain kind. Therefore, *Food*, *Painting*, *Face* and *House* tasks were dedicated to “food items”, “paintings”, “human faces” and “houses”, respectively.

All tasks were organized as a block-design experiment with one condition per trial. Every trial started with a fixation cross, whose duration was jittered between 0.5 seconds and 4.5 seconds, after which a picture of an item was displayed on the screen together with a rating scale and a cursor. Participants were to indicate how pleasant the presented stimulus was, by sliding the cursor along the scale. Such scale ranged between 1 and 100. The value 1 corresponded to the choices “unpleasant” or “indifferent”; the middle of the scale corresponded to the choice “pleasant”; and the value 100 corresponded to the choice “very pleasant”. Therefore, the ratings related only to the estimation of the positive-incentive value of the items displayed.

One full session was dedicated to the data collection of all tasks. It comprised eight runs with sixty trials each. Although each trial had a variable duration, according to the time spent by the participant in the assessment, no run lasted longer than eight minutes and sixteen seconds. Every task was presented twice in two fully dedicated runs. The stimuli were always different between runs of the same task. As a consequence, no stimulus was ever repeated in any trial and, thus, no item was ever assessed more than once by the participants. To avoid any selection bias in the sequence of stimuli, the order of their presentation was shuffled across trials and between runs of the same type. This shuffle is embedded in the code of the protocol and, thus, the sequence was determined upon launching it. Consequently, the sequence of stimuli was also random across subjects. For each run (of each session), this sequence was properly registered in the logfile generated by the protocol.

### *Theory-of-mind* and *pain matrices* task battery

This battery of tasks was adapted from the original task-fMRI localizers of Saxe Lab, intended to identify functional regions-of-interest in the *Theory-of-Mind* network and *Pain Matrix* regions. These localizers rely on a set of protocols along with verbal and non-verbal stimuli, whose material was obtained from https://saxelab.mit.edu/localizers.

Minor changes were employed in the present versions of the tasks herein described. Because the cohort of this dataset is composed solely of native French speakers, the verbal stimuli were thus translated to French. Therefore, the durations of the reading period and the response period within conditions were slightly increased.

#### Theory-of-mind localizer (TOM localizer)

The *Theory-of-Mind Localizer* (TOM localizer) was intended to identify brain regions involved in theory-of-mind and social cognition, by contrasting activation during two distinct story conditions: *(1) belief judgments*, reading a *false-belief* story that portrayed characters with false beliefs about their own reality; and *(2) fact judgments*, reading a story about a false photograph, map or sign^28^.

The task was organized as a block-design experiment with one condition per trial. Every trial started with a fixation cross of twelve seconds, followed by the main condition that comprised a reading period of eighteen seconds and a response period of six seconds. Its total duration amounted to thirty six seconds. There were ten trials in a run, followed by an extra-period of fixation cross for twelve seconds at the end of the run. Two runs were dedicated to this task in one single session.

The designs, i.e. the sequence of conditions across trials, for two possible runs were pre-determined by the authors of the original study and *hard-coded* in the original protocol (see Section “Theory-of-Mind and Pain Matrices task battery”). The IBC-adapted protocols contain the exactly same designs. For all subjects, design #1 was employed for the PA-run and design #2 for the AP-run.

### *Theory-of-mind* and *pain-matrix narrative localizer* (Emotional Pain localizer)

The *Theory-of-Mind and Pain-Matrix Narrative Localizer* (Emotional Pain localizer) was intended to identify brain regions involved in theory-of-mind and Pain Matrix areas, by contrasting activation during two distinct story conditions: reading a story that portrayed characters suffering from *(1) emotional pain* and *(2) physical pain*^29^.

The experimental design of this task is identical to the one employed for the TOM localizer, except that the reading period lasted twelve seconds instead of eighteen seconds. Two different designs were pre-determined by the authors of the original study and they were employed across runs and participants, also in the same way as described for the TOM localizer (see Section “Theory-of-Mind Localizer (TOM localizer)”).

### *Theory-of-mind* and *pain matrix movie localizer* (Pain Movie localizer)

The *Theory-of-Mind and Pain Matrix Movie Localizer* (Pain Movie localizer) consisted in the display of “Partly Cloud”, a 6-minute movie from Disney Pixar, in order to study the responses implicated in theory-of-mind and pain-matrix brain regions^29,30^.

Two main conditions were thus hand-coded in the movie, according to^30^, as follows: *(1) mental movie*, in which characters were experiencing changes in beliefs, desires, and/or emotions; and *(2) physical pain movie*, in which characters were experiencing physical pain. Such conditions were intended to evoke brain responses from theory-of-mind and pain-matrix networks, respectively. All moments in the movie not focused on the direct interaction of the main characters were considered as a baseline period.

### *Visual short-term memory* (VSTM) and *enumeration* task battery

This battery of tasks was adapted from the control experiment described in^31^. They were intended to investigate the role of the Posterior Parietal Cortex (PPC) involved in the concurrent processing of a variable number of items. Because subjects can only process three or four items at a time, this phenomenon may reflect a general mechanism of object individuation^34,35^. On the other hand, PPC has been implicated in studies of capacity limits, during *Visual Short-Term Memory* (VSTM)^36^ and *Enumeration*^35^. While the former requires high encoding precision of items due to their multiple features, like location and orientation, the latter requires no encoding of object features. By comparing the neural response of the PPC with respect to the two tasks, the original study demonstrated a non-linear increase of activation, in this region, along with the increasing number of items. Besides, this relationship was different in the two tasks. Concretely, PPC activation started to increase from two items onward in the VSTM task, whereas such increase only happened from three items onward in the Enumeration task.

For both tasks, the stimuli consisted of sets of tilted dark-gray bars displayed on a light-gray background. Additionally, minor changes were employed in their present versions herein described: *(1)* both the response period and the period of the fixation dot at the end of each trial were made constant in both tasks; and *(2)* for the Enumeration task, answers were registered via a button-press response box instead of an audio registration of oral responses as in the original study.

### Visual short-term memory task (VSTM)

In the VSTM task, participants were presented with a certain number of bars, varying from one to six.

Every trial started with the presentation of a black fixation dot in the center of the screen for 0.5 seconds. While still on the screen, the black fixation dot was then displayed together with a certain number of tilted bars –variable between trials from one to six– for 0.15 seconds. Afterwards, a white fixation dot was shown for 1 second. It was next replaced by the presentation of the test stimulus for 1.7 seconds, displaying identical number of tilted bars in identical positions together with a green fixation dot. The participants were to remember the orientation of the bars from the previous sample, and answer with one of the two possible button presses, depending on whether one of the bars in the current display had changed orientation by 90 °, which was the case in half of the trials. The test display was replaced by another black fixation dot for a fixed duration of 3.8 seconds. Thus, the trial was 7.15 seconds long. There were 72 trials in a run and four runs in one single session. Pairs of runs were launched consecutively. To avoid selection bias in the sequence of stimuli, the order of the trials was shuffled according to numerosity and change of orientation within runs and across participants.

### Enumeration task

In the Enumeration task, participants were presented with a certain number of bars, varying from one to eight.

Every trial started with the presentation of a black fixation dot in the center of the screen for 0.5 seconds. While still on the screen, the black fixation dot was then displayed together with a certain number of tilted bars –variable between trials from one to eight– for 0.15 seconds. It was followed by a response period of 1.7 s, in which only a green fixation dot was being displayed on the screen. The participants were to remember the number of the bars that were shown right before and answer accordingly, by pressing the corresponding button. Afterwards, another black fixation dot was displayed for a fixed duration of 7.8 seconds. The trial length was thus 9.95 seconds. There were ninety six trials in a run and two (consecutive) runs in one single session. To avoid selection bias in the sequence of stimuli, the order of the trials was shuffled according to numerosity within runs and across participants.

### *Self* task

The *Self* task was adapted from the study^32^, originally developed to investigate the *Self-Reference Effect* in older adults. This effect pertains to the encoding mechanism of information referring to the self, characterized as a memory-advantaged process. Consequently, memory-retrieval performance is also better for information encoded in reference to the self than to other people, objects or concepts.

The present task was thus composed of two phases, each of them relying on encoding and recognition procedures. The encoding phase was intended to map brain regions related to the encoding of items in reference to the self, whereas the recognition one was conceived to isolate the memory network specifically involved in the retrieval of those items. The phases were interspersed, so that the recognition phase was always related to the encoding phase presented immediately before.

The encoding phase had two blocks. Each block was composed of a set of trials pertaining to the same condition. For both conditions, a different adjective was presented at every trial on the screen. The participants were to judge whether or not the adjective described themselves –*self-reference encoding* condition– or another person –*other-reference encoding* condition. The other person was a public figure in France around the same age range as the cohort, whose gender matched the gender of every participant. Two public figures were mentioned, one at the time, across all runs; four public figures –two of each gender– were selected beforehand. By this way, we ensured that all participants were able to successfully characterize the same individuals, holding equal the levels of familiarity and affective attributes with respect to these individuals.

In the recognition phase, participants were to remember whether or not the adjectives had also been displayed during the previous encoding phase. This phase was composed of a single block of trials, pertaining to three categories of conditions. *New* adjectives were presented during one half of the trials whereas the other half were in reference to the adjectives displayed in the previous phase. Thus, trials referring to the adjectives from “self-reference encoding” were part of the *self-reference recognition* category and trials referring to the “other-reference encoding” were part of the *other-reference recognition* category. Conditions were then defined according to the type of answer provided by the participant for each of these categories (see Online-only Table 1 for details).

There were four runs in one session. The first three ones had three phases; the fourth and last run had four phases (see Table 2). Their total durations were twelve and 15.97 seconds, respectively. Blocks of both phases started with an *instruction* condition of five seconds, containing a visual cue. The cue was related to the judgment that should be performed next, according to the type of condition featured in that block. A set of trials, showing different adjectives, were presented afterwards. Each trial had a duration of five seconds, in which a response was to be provided by the participant. During the trials of the encoding blocks, participants had to press the button with their left or right hand, depending on whether they believed or not the adjective on display described someone (i.e. self or other, respectively for “self-reference encoding” or “other-reference encoding” conditions). During the trials of the recognition block, participants had to answer in the same way, depending on whether they believed or not the adjective had been presented before. A fixation cross was always presented between trials, whose duration was jittered between 0.3 seconds and 0.5 seconds. A rest period was introduced between encoding and recognition phases, whose duration was also jittered between ten and fourteen seconds. Long intervals between these two phases, i.e. longer than ten seconds, ensured the measurement of long-term memory processes during the recognition phase, at the age range of the cohort^37,38^. Fixation-cross periods of three and fifteen seconds were also introduced in the beginning and end of each run, respectively.

All adjectives were presented in the lexical form according to the gender of the participant. There were also two sets of adjectives. One set was presented as new adjectives during the recognition phase and the other set for all remaining conditions of both phases. To avoid cognitive bias across the cohort, sets were switched for the other half of the participants. Plus, adjectives never repeated across runs but their sequence was fixed for the same runs and across participants from the same set. Yet, pseudo-randomization of the trials for the recognition phase was pre-determined by the authors of the original study, according to their category (i.e. “self-reference recognition”, “other-reference recognition” or “new”), such that no more than three consecutive trials of the same category were presented within a block.

For sake of clarity, Online-only Table 1 contains a full description of all main conditions employed in the experimental design of this task.

### Bang task

The *Bang* task was adapted from the study^33^, dedicated to investigate aging effects on neural responsiveness during naturalistic viewing.

The task relies on watching –viewing and listening– of an edited version of the episode “Bang! You’re Dead” from the TV series “Alfred Hitchcock Presents”. The original black-and-white, 25-minute episode was condensed to seven minutes and fifty five seconds while preserving its narrative. The plot of the final movie includes scenes with characters talking to each other as well as scenes with no verbal communication. Conditions of this task were thus set by contiguous scenes of speech and no speech.

This task was performed during a single run in one unique session. Participants were never informed of the title of the movie before the end of the session. Ten seconds of acquisition were added at the end of the run. The total duration of the run was thus eight minutes and five seconds.

## Data Acquisition

**Fig. 1:**
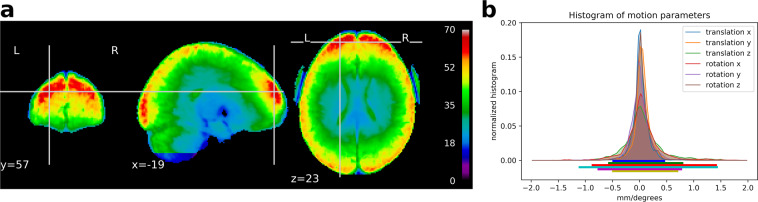
Global quality indices of the acquired data: tSNR map and motion magnitude distribution. (**a**) The tSNR map displays the average of tSNR across all tasks and subjects. This shows values mostly between 30 and 70, with larger tSNR in cortical regions, except for the bottom of the cerebellum, that was acquired at a lesser extent in this data release. (**b**) Density of within-run motion parameters, pooled across subjects and tasks. Six distributions are plotted, for the six rigid-body parameter of head motion (translations and rotations are in mm and degrees, respectively). Each distribution is based on ∼101*k* EPI volumes of 11 subjects, corresponding to all time frames for all acquisitions and subjects. Bold lines below indicate the 99% coverage of all distributions and show that motion parameters mostly remain confined to 1.5 mm/1degree across 99% of all acquired images.

Data across participants were acquired throughout six MRI sessions, whose structure is described in Table 2. Deviations from this structure were registered for two MRI sessions. Besides and as referred in Section “Data quality” as well as Fig. 1, a drop of the tSNR was identified for some MRI sessions. Additionally, data of the tasks featuring this release were not yet collected for subject 8 (consult Section “Participants” for further details). These anomalies in the data are summarized on Online-only Table 2.

### Behavioral Data

Active responses were required from the participants in all tasks. The registry of all behavioral data, such as the qualitative responses to different conditions and corresponding response times, was held in log files generated by the stimulus-delivery software.

### Imaging Data

FMRI data were collected using a *Gradient-Echo* (GE) pulse, whole-brain *Multi-Band* (MB) accelerated^39,40^ *Echo-Planar Imaging* (EPI) T2*-weighted sequence with *Blood-Oxygenation-Level-Dependent* (BOLD) contrasts. Two different acquisitions for the same run were always performed using two opposite phase-encoding directions: one from *Posterior to Anterior* (PA) and the other from *Anterior to Posterior* (AP). The main purpose was to ensure within-subject replication of the same tasks, while mitigating potential limitations concerning the distortion-correction procedure.

*Spin-Echo* (SE) EPI-2D image volumes were acquired in order to compensate for spatial distortions. Similarly to the GE-EPI sequences, two different phase-encoding directions, i.e. PA and AP, were employed in different runs pertaining to this sequence. There were four runs per session: one pair of PA and AP SE EPI-2D before the start of the GE-EPI sequences and another pair at the end.

The parameters for all types of sequences employed are provided in^19^ as well as in the documentation available on the IBC website: https://project.inria.fr/IBC/data/

## Data Analysis

### Image conversion

The acquired DICOM images were converted to NIfTI format using the dcm2nii tool, which can be found at https://www.nitrc.org/projects/dcm2nii. During conversion to NIfTI, all images were fully anonymized, i.e. pseudonyms were removed and images were defaced using the mri_deface command line from the Freesurfer-6.0.0 library.

### Preprocessing

Source data were preprocessed using the same pipeline employed for the first release of the IBC dataset. Thus, refer to^19^ for more complete information about procedures undertaken during this stage.

In summary, raw data were preprocessed using *PyPreprocess*

(https://github.com/neurospin/pypreprocess), dedicated to launch in the python ecosystem pre-compiled functions of *SPM12* software package v6685 and *FSL* library v5.0.

Firstly, susceptibility-induced off-resonance field was estimated from four SE EPI-2D volumes, each half acquired in opposite phase-encoding directions (see Section “Imaging Data” for details). The images were corrected based on the estimated deformation model, using the *topup* tool^41^ implemented in FSL^42^.

GE-EPI volumes of each participant were then aligned to each other, using a rigid body transformation, in which the average volume of all images across runs (per session) was used as reference^43^.

The mean EPI volume was also co-registered onto the T1-weighted MPRAGE (anatomical) volume of the corresponding participant^44^, acquired during the *Screening* session (consult^19^ for details).

The individual anatomical volumes were then segmented into tissue types in order to allow for the normalization of both anatomical and functional data into the standard MNI152 space, which was performed using the *Unified Segmentation* probabilistic framework^45^. Concretely, the segmented volumes were used to compute the deformation field for normalization to the standard MNI152 space.

### FMRI model specification

FMRI data were analyzed using the General Linear Model (GLM). Regressors-of-interest in the model were designed to capture variations in BOLD signal, which are in turn coupled to neuronal activity pertaining to task performance. To this end, the temporal profile of task stimuli is convolved with the Hemodynamic Response Function (HRF) –defined according to^46,47^– in order to obtain the theoretical neurophysiological profile of brain activity in response to behavior. The temporal profiles of stimuli, for block-design experiments, are typically characterized by boxcar functions defined by triplets –onset time, duration and trial type– that can be extracted from log files’ registries generated by the stimulus-delivery software.

Because the present release encompasses tasks with different types of experimental designs, regressors-of-interest can refer to either conditions, wherein main effects of stimuli span a relatively long period, or parametric effects of those stimuli. Online-only Table 1 contains a complete description of all regressors-of-interest implemented in the models of every task.

Nuisance regressors were also modeled in order to account for different types of spurious effects arising during acquisition time, such as fluctuations due to latency in the HRF peak response, movements, physiological noise and slow drifts within run. We also account for another type of regressors-of-no-interest, referring to either no responses or non-correct behavioral responses, implemented in the model of the Self task. Concretely, the regressors “encode_self_no_response”, “encode_other_no_response”,

“recognition_self_no_response” and “recognition_other_no_response” –related to absence of responses in each condition– plus “recognition_self_miss” and “recognition_other_miss” –related to the unsuccessful recognition of an adjective previously presented– as well as “false_alarm” –related to the misrecognition of a new adjective as one already presented– were modeled separately as means to grant an accurate isolation of the effects pertaining to “recognition” and “self reference” in the regressors-of-interest (see Section “Self task” for further details about the design of this task).

A complete description of the general procedures concerned with the GLM implementation of the IBC data can be found in the first data-descriptor article^19^. Such implementation was performed using *Nistats* python module v0.0.1b (https://nistats.github.io), leveraging *Nilearn* python module v0.6.0^48^ (https://nilearn.github.io/).

### Model estimation

In order to restrict GLM parameters estimation to voxels inside functional brain regions, a brain mask was extracted from the normalized mean GE-EPI volume thresholded at a liberal level of 0.25, using Nilearn. This corresponds to a 25% average probability of finding gray matter in a particular voxel across subjects. A mass-univariate GLM fit was then applied to the preprocessed EPI data, for every run in every task, using Nistats. For this fit, we set a spatial smoothing of 5 mm full-width-at-half-maximum as a regularization term of the model; spatial smoothing is a standard procedure that ensures an increase of the *Signal-to-Noise Ratio* (SNR) at the same time that facilitates between-subject comparison. Parameter estimates for all regressors implemented in the model were computed, along with the respective covariance, at every voxel. Linear combinations between parameter estimates computed for the regressors-of-interest (listed on Online-only Table 1) as well as for the baseline were performed in order to obtain contrast maps with the relevant evoked responses.

More details about model estimation can be found in the first data-descriptor article^19^. Its implementation and the ensuing statistical analyses were performed using Nistats (about Nistats, see Section “FMRI Model Specification”).

### Summary statistics

Because data were collected per task and subject in –at least– two acquisitions with opposite phase-encoding directions (see Section “Imaging Data” for details), statistics of their joint effects were calculated under a *Fixed-Effects* (FFX) model.

*t*-tests were then computed at every voxel for each individual contrast, in order to assess for statistical significance in differences among evoked responses. To assure standardized results that are independent from the number of observations, *t*-values were directly converted into *z*-values.

Data derivatives are thus delivered as individual contrast maps containing standard scores in all voxels that are confined to a grey-matter-mask with an average threshold >25% across subjects. We note that these postprocessed individual maps were obtained from the GLM fit of preprocessed EPI maps and, thus, they are represented in the standard MNI152 space. For more information about the access to the data derivatives, refer to Section “Derived statistical maps”.

## Data Records

Both raw fMRI data (*aka* “source data”) as well as derived statistical maps of the IBC dataset are publicly available.

### Source data

Source data of the present release (plus first and third releases) can be accessed via the public repository *OpenNeuro*^49^ under the data accession number *ds*002685^50^. This collection comprises ∼1.1 TB of MRI data. A former collection only referring to source data from the first release is still available in the same repository^51^.

The NIfTI files as well as paradigm descriptors and imaging parameters are organized per run for each session according to BIDS Specification:

- the data repository is organized in twelve main directories sub-01 to sub-15; we underline that sub-02, sub-03 and sub-10 are not part of the dataset and corresponding data from sub-08 will be made available in further releases (see Table 1);
- data from each subject are numbered on a per-session basis, following the chronological order of the acquisitions; we also note that this order is not the same for all subjects; the IBC documentation can be consulted for the exact correspondence between session number and session id for every subject on https://project.inria.fr/IBC/data/ (session id’s of the first and second releases are respectively provided on Table 2 of ^19^ and Table 2 of the present article);
- acquisitions are organized within session by modality;
- different identifiers are assigned to different types of data as follows:
  - gzipped NIfTI 4D image volumes of BOLD fMRI data are named as sub-XX_ses-YY_task-ZZZ_dir-AA_bold.nii.gz, in which XX and YY refer respectively to the subject and session id, ZZZ refers to the name of the task, and AA can be either ‘PA’ or ‘AP’ depending on the phase-encoding direction;
  - event files are named as sub-XX_ses-YY_task-ZZZ_dir-AA_event.tsv;
  - single-band, reference images are named as sub-XX_ses-YY_task-ZZZ_dir-AA_sbref.nii.gz.

Although BIDS v1.2.1 does not provide support for data derivatives, a similar directory tree structure was still preserved for this content.

### Derived statistical maps

The unthresholded-statistic, contrast maps have been released in the public repository *NeuroVault*^52^ with the *id* = 6618^53^. This collection comprises data from both releases. A former collection only referring to data derivatives from the first release is still available in the same repository (*id* = 4438^54^).

## Technical Validation

### Behavioral data

Response accuracy of behavioral performance was calculated for those tasks requiring overt responses. It aims at providing a quantitative assessment of the quality of the imaging data in terms of subjects’ compliance. Because imaging data reflect herein brain activity related to behavior, scores of response accuracy across trials are good indicators of faithful functional representations regarding the cognitive mechanisms involved in the correct performance of the task. Individual scores are provided as percentages of correct responses with respect to the total number of responses in every run of a given task. The average of these scores is also provided as an indicator of the overall performance of the participant for that specific task.

### Mental time travel task battery

Scores for MTT WE and MTT SN tasks are provided on Online-only Table 3. Participants were to give one answer out of two possible answers during the Response condition (see Section “Mental Time Travel (MTT) task battery” for details); one of the answers was the correct one. Nevertheless, trials were composed of one series of four consecutive Events interspersed by the corresponding Response. As a result, participants sometimes anticipated or delayed their answers when they were still listening the action during the corresponding Event condition or already in the next Event condition, respectively. To account for correct answers provided under these circumstances, responses given during the first half of an Event (except for the first one of the series) were considered as answers pertaining to the previous Event; on the other hand, responses given during the second half of an Event were considered as answers pertaining to the current Event. The average ± SD across participants for the two tasks are 76 ± 13% and, thus, higher than chance level (50%). We conclude that participants not only learnt correctly the stories plotted in both tasks but also performed successfully the time and space shifts upon listening the Event conditions.

#### Theory-of-mind

In the Theory-of-Mind task, participants were to read stories involving either false-beliefs about the state of the world or scenery representations that were misleading or outdated. Afterwards they were to answer a question pertaining to the plot, in which one out of two possible answers was correct (see Section “Theory-of-Mind Localizer (TOM localizer)” for details). Online-only Table 4 provides the individual scores achieved for this task. The average ± SD across participants are 74 ± 16%, i.e. higher than chance level (50%). These results show that overall the participants understood the storylines and thus they were able to successfully judge the facts pointed out in the questions.

### Visual short-term memory task

**Fig. 2:**
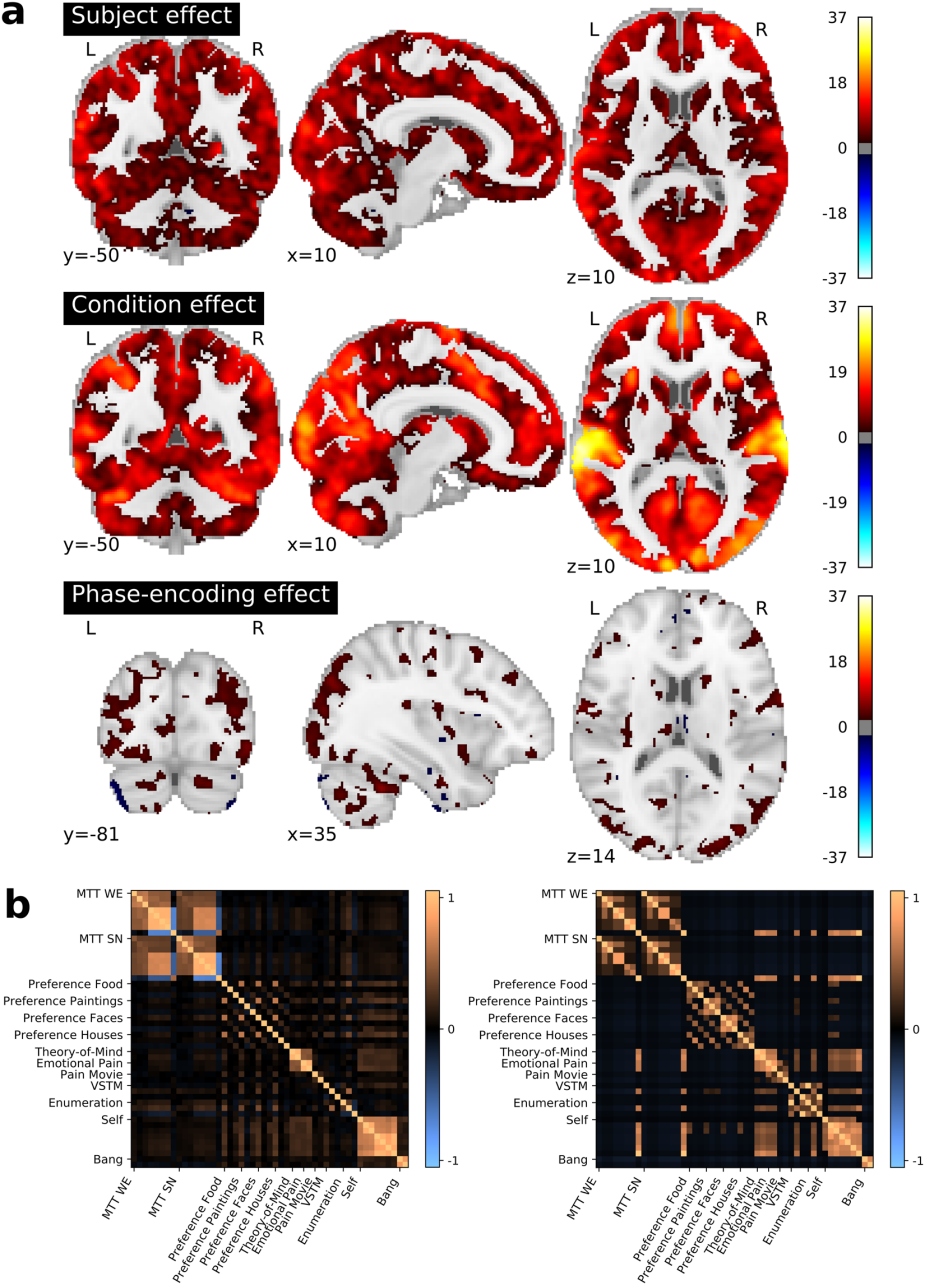
Overview of the information conveyed by the activation maps resulting from a first-level analysis. (**a**) Effects of subject, experimental condition and phase-encoding direction. A per-voxel ANOVA breaks the variance of the set of brain maps into subject, experimental condition, and phase-encoding direction related effects. All maps are given in *z*-scale and thresholded at an FDR level of 0.05. We note that these results strictly follow the gray-matter structure, as an anatomically-defined gray-matter mask was used in the first-level GLM model (see Section “Model Estimation”). (**b**) The similarity between condition-related activation maps, averaged across subjects (left), is related to the similarity of the same conditions, when these are characterized in terms of the Cognitive Atlas (right).

In the VSTM task, participants were asked to identify, for every trial, whether there had been a change in the orientation of one of the bars during two consecutive displays of the same number of bars (see Section “Visual Short-Term Memory task (VSTM)” for details). There were thus two possible answers. Online-only Table 5 provides the individual scores for every run and the average across runs, grouped by numerosity of the visual stimuli (measured by the number of bars); numerosity ranged from one to six. In line with the behavioral results reported in the original study (Fig. 2 - plot E of ^31^), the scores start decreasing more prominently for *numerosity* >3 (see Online-only Table 6).

### Enumeration task

In the Enumeration task, participants were asked to identify, for every trial, the exact number of bars displayed on the screen (see Section “Enumeration task” for details). The number of bars ranged from one to eight; there were thus eight possible answers. Online-only Table 7 provides the individual scores for every run and the average across runs, grouped by numerosity of the visual stimuli (measured by the number of bars). Following the overall trend of the behavioral results reported in the original study (Fig. 2 - plot D of^31^), the scores decrease substantially for *numerosity* >4 (see Online-only Table 8).

### Self task – recognition phase

The Self-task paradigm comprised two different phases: the encoding and recognition phases (see Section “Self task” for details). Both of them pertained to overt responses, although only the recognition phase required correct answers. In this particular phase, participants were to judge whether the adjective under display had already been presented in the previous encoding phase. Online-only Table 9 provides the individual scores for every run and the average across runs. The average ± SD across participants are 83 ± 8%, i.e. higher than chance level (50%), showing that participants successfully recognized either familiar or new adjectives in the majority of the “recognition” trials. Despite some low behavioral scores registered (particularly in run 3 for participant 1 and runs 0 and 1 for participant 5), we have only included trials with active and correct responses in the regressors-of-interest and, thus, neuroimaging results are not impacted by spurious effects potentially derived from occasional poor performances.

## Imaging Data

### Data quality

In order to provide an approximate estimate of data quality, measurements of the preprocessed data are presented in Fig. 1 and described as follows:

- The temporal SNR (tSNR), defined as the mean of each voxels’ time course divided by their standard deviation, on normalized and unsmoothed data averaged across all acquisitions. Its values go up to 70 in the cortex. Given the high resolution of the data (1.5 mm isotropic), such values are indicative of a good image quality^55^;
- The histogram of the six rigid body motion estimates of the brain per scan, in mm and degrees (for translational and rotational motion, respectively), together with their 99% coverage interval. One can notice that this interval ranges approximately within [−1.1, 1.5] mm as well as degrees, showing that motion excursions beyond 1.5 mm or degrees are rare. No acquisition was discarded due to excessive motion (>2 mm or degrees).

**Fig. 3:**
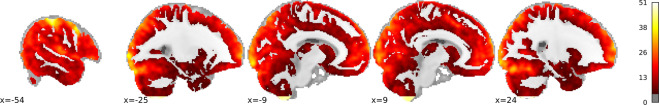
Brain coverage of the IBC-dataset second-release. Group-level F-map, at a threshold of *p* < 0.05, Bonferroni-corrected, representing the total area of the brain significantly covered by the tasks featuring solely the second release of the IBC dataset (FFX across tasks and subjects). One can easily observe an extensive brain coverage, with higher effects in lateral cortical areas by comparison with medial cortical areas and sub-cortical areas. We note that these results strictly follow the gray-matter structure, as an anatomically-defined gray-matter mask was used in the first-level GLM model (see Section “Model Estimation”).

We observe an overall improvement of the tSNR with respect to the first release (see Section “Data quality” and Fig. 1 in^19^) in cortical regions, wherein functional activity in response to behavior is greatly covered (see Fig. 3). However, a poor coverage was attained for the bottom of the cerebellum (for more details about what sessions contributed specifically for this tSNR deviation, consult Online-only Table 2). Yet, as shown by Fig. 2, this issue is not driven by subject, condition or phase-encoding direction effects. It refers instead to a transient irregularity that affected the field-of-view during the acquisition period.

### Relevance of the IBC dataset for brain mapping

#### Effect of subject identity and task stimuli on activation

Taking into account the output of the GLM analysis for each acquisition, an assessment was performed at every voxel concerning how much variability of the signal could be explained by the effect of: *(1)* subject identity, *(2)* condition, and *(3)* phase-encoding direction. To assess the impact of these three factors, a one-way *Analysis-of-Variance* (ANOVA) of all contrast maps –1782 maps from the 11 subjects– was computed and results from the first-level analysis of the data were obtained for the aforementioned factors. The resulting statistical maps are displayed on the top of the Fig. 2. They show that both subject and condition effects are uniformly significant at *p* < 0.05, corrected for multiple comparisons using *False Discovery Rate* (FDR). Condition effects are overall higher than subject effects, particularly in sensory cortices like visual, auditory and somato-sensory regions. Effects pertaining to the phase-encoding direction are only significant in smaller areas comprising superior cortical regions, with special emphasis in the occipital lobe. We hypothesize that such results derive from the fact that PA data accounts for a larger amount than AP data in this dataset (see Table 2).

#### Similarity of brain activation patterns fits between-task similarity

Within-subject correlation matrices of all FFX contrast-maps pertaining to experimental conditions *vs*. baseline were computed as means to summarize the similarity between the functional responses to these conditions. The average of the correlation matrices across subjects was then estimated in order to assess the pattern of similarity between tasks. Because this second release accounts for a total of 49 conditions (and, consequently, 49 elementary contrasts) among all tasks, the average of correlation matrices from all participants is represented as a 49 × 49 correlation matrix (see Fig. 2, bottom-left). Note that this approach is different from performing a second-level analysis across subjects per task and compute the corresponding correlation matrix between tasks. Besides, experimental conditions were also encoded according to the Cognitive Atlas’ ontology (https://www.cognitiveatlas.org, see also^21^ for the link between each condition and cognitive labels). This labeling is listed in detail on Online-only Table 10, accounting for a total of 59 different cognitive components shared by the elementary contrasts. (Note that the number of cognitive components for the total amount of contrasts is 63 and, thus, slightly higher than the amount reported for the elementary contrasts only.) The 49 × 49 correlation matrix of the conditions defined in terms of occurrences of these cognitive descriptions was computed, since such labeling offers an approximate characterization of the tasks (see Fig. 2, bottom-right). They show clear similarities, together with discrepancies that are worthy of further investigation.

In order to assess the feasibility of our cognitive descriptions, we then tested how similar the cross-condition characterizations using the cognitive labels and the activation maps are from each other. A *Representational Similarity Analysis*^56^ was performed between the activation-map and the cognitive-occurrence correlation matrices by means of the Spearman correlation between their upper-triangular coefficients, which amounted to 0.21 with $p\le 1{0}^{-13}$. We then repeated this analysis for tasks of the first and second releases all together. They both combine 109 elementary conditions with 106 cognitive components. The Spearman correlation between the two different matrices is, in this case, 0.23 with $p\le 1{0}^{-72}$, which is not only higher than the correlation obtained from the tasks of the second release but also from those of the first release (Spearman correlation: 0.21, with $p\le 1{0}^{-17}$)^19^. We conclude that similarity tends to increase as more data is included in the dataset because new cognitive elements are added to the description of all tasks.

### Brain coverage

Figure 3 displays all brain areas significantly covered by the tasks of this dataset extension. Most cortical and sub-cortical regions are altogether represented by this group of tasks. By comparison with the first release, the present one shows a prominent improvement of significant coverage in the cingulate cortex, namely its posterior section in the parietal lobe and its anterior section in the prefrontal lobe. Similarly, a better coverage of the right anterior temporal lobe can also be observed in this release.

On the other hand, a prominent deviation of the results at the bottom of the cerebellum in comparison with its neighboring regions is present due to a lesser coverage of this area, as reported in Section “Data quality” and Fig. 1.

As the dataset becomes larger, one may expect a progressively improvement of brain coverage. However, as referred already in^19^, this coverage may not be fully attained due to MR-related technical restrictions. Some locations are particularly sensitive to e.g. coil sensitivity or intra-voxel dephasing, which can result in a reduced tSNR in certain functional brain regions.

## Usage Notes

The IBC project keeps promoting the open access and encouraging the community in adhering to practices concerned with data sharing and reproducibility in neuroimaging. Thus, free online access of raw data and derivatives is respectively assured by *OpenNeuro*^50^ and *NeuroVault*^53^.

This second release, along with the first one, brings together a variety of tasks –featuring in total 221 independent contrasts– allowing at the same time for an increase of the brain area significantly covered by these tasks (see Section “Brain Coverage” for more details).

The collection of new data continues till year 2022 and more releases are expected in the next years. The third release is already planned for the present year and it will be fully dedicated to the visual system; it will contain tasks pertaining to retinotopy, movie watching and viewing of naturalistic scenes. Future releases will also address in greater depth the auditory system through tonotopy as well as tasks on auditory language comprehension, listening of naturalistic sounds and music perception. Other tasks on biological motion, stimulus salience, working memory, motor inhibition, risk-based decision making and spatial navigation will also integrate these future releases. Finally, although IBC stands foremost as a task-fMRI dataset with a strong emphasis in the task dimension, future releases will be also dedicated to resting-state fMRI data as well as to other MRI modalities, concretely high-resolution T1- and T2- weighted, diffusion-weighted, T1- and T2- relaxometry and myelin water fraction.

The official website of the IBC project (https://project.inria.fr/IBC/) can be consulted anytime for a continuous update about the latest and forthcoming releases.

## Data Availability

Metadata concerning the stimuli presented during the BOLD fMRI runs are publicly available at https://github.com/hbp-brain-charting/public_protocols. They include: *(1)* the task-stimuli protocols; *(2)* demo presentations of the tasks as video annotations; *(3)* instructions to the participants; and *(4)* scripts to extract paradigm descriptors from log files for the GLM estimation. Task-stimuli protocols from Preference, TOM and VSTM + Enumeration batteries were adapted from the original studies in order to comply with the IBC experimental settings, without affecting the design of the original paradigms. MTT battery pertains to an original protocol developed in Python under the context of the IBC project. Protocols of Self and Bang tasks were re-written from scratch in Python with no change of the design referring to the original paradigms. The scripts used for data analysis are available on GitHub under the Simplified BSD license: https://github.com/hbp-brain-charting/public_analysis_code. Additionally, a full description of all contrasts featuring data derivatives (see Section “Derived statistical maps” for details) as well as a list of the main contrasts are also provided under the folder hbp-brain-charting/public_analysis_code/ibc_data.

## Online-only Tables

**Online-only Table 1:** Regressors-of-Interest implemented in the design matrices of the tasks for the main contrasts of this dataset release.

Regressor-of-Interest	Description of the effect modeled
**MTT task battery**^*****^	
we/sn_average_reference	action in the story to serve as reference for the time or space judgment in the same trial in the west-east/south-north island
we/sn_all_space_cue	cue indicating a question about spatial orientation in the west-east/south-north island
we/sn_all_time_cue	cue indicating a question about time orientation in the west-east/south-north island
westside/southside_event	action to be judged whether it takes place west/south or east/north from this reference, that actually takes place west/south from this reference
eastside/northside_event	action to be judged whether it takes place west/south or east/north from this reference, that actually takes place east/north from this reference
we/sn_before_event	action to be judged whether it takes place before or after this reference, that actually takes place before this reference, in the west-east/south-north island
we/sn_after_event	action to be judged whether it takes place before or after this reference, that actually takes place after this reference, in the west-east/south-north island
we/sn_all_event_response	motor responses performed after every event condition in the west-east/south-north island
**Preference task battery**^**§**^	
preference_constant	main effect of condition concerning the classification of the level of pleasantness of an item displayed on the screen
preference_linear	parametric effect concerning the rating provided by the participant
preference_quadratic	parametric effect of the squared rating
**TOM localizer**	
belief	main effect of condition concerning the reading of a *false-belief* story, that portrayed characters with false beliefs about their own reality
photo	main effect of condition concerning the reading of a story related to a false photograph, map or sign
**Emotional Pain localizer**	
emotional_pain	main effect of condition concerning the reading of a story that portrayed characters suffering from emotional pain
physical_pain	main effect of condition concerning the reading of a story that portrayed characters suffering from physical pain
**Pain Movie localizer**	
movie_mental	main effect of condition concerned with watching a movie scene whose characters experience changes in beliefs, desires and/or emotions
movie_pain	main effect of condition concerned with watching a movie scene whose characters experience physical pain
**VSTM task**	
vstm_constant	main effect of condition concerned with judging whether any bar changed orientation within two consecutive displays of bar sets on the screen
vstm_linear	parametric effect of the number of bars displayed during two consecutive displays^†^
vstm_quadratic	quadratic effect of the number of bars
**Enumeration task**	
enumeration_constant	main effect of condition concerned with judging the number of bars displayed on the screen
enumeration_linear	parametric effect of the number of bars displayed
enumeration_quadratic	quadratic effect of the number of bars
**Self task**	
instruction	main effect of condition concerning the presentation of a question related to the succeeding block^‡^
encode_self^1^	main effect of condition concerned with judging whether a certain adjective —displayed on the screen— qualifies oneself
encode_other^1^	main effect of condition concerned with judging whether a certain adjective —displayed on the screen— qualifies someone else
recognition_self_hit^2^	main effect of condition concerning the successful recognition of an adjective —displayed on the screen— as having been already presented during one “encode_self” trial of the preceding encoding phase
recognition_other_hit^2^	main effect of condition concerning the successful recognition of an adjective —displayed on the screen— as having been already presented during one “encode_other” trial of the preceding encoding phase
correct_rejection^2^	main effect of condition concerning the successful identification that a *new* adjective has never been presented before
**Bang task**	
talk	main effect of condition concerned with watching contiguous scenes of speech
no_talk	main effect of condition concerned with watching contiguous scenes of non-speech

*MTT-task battery comprises two tasks that differ in their cardinal-orientation judgment –West-East and South-North– as detailed in Section “Mental Time Travel (MTT) task battery”. The experimental paradigm is the same and regressors-of-interest are equivalent with respect to cardinality.

$\dagger$In every trial, the same number of bars was kept between the two consecutive displays.

${\rm{\ddagger }}$Questions were according to the type of the succeeding block. Thus, “encode_self” blocks were preceded by the question *“Are you?”*; “encode_other” blocks were preceded by the question *“Is < name_of_famous_person > ?”*; and recognition-phase blocks were preceded by the question *“Have you seen?”*.

^1^Regressor modeling condition from the Encoding phase.

^2^Regressor modeling condition from the Recognition phase.

**Online-only Table 2:** Data anomalies.

	Participants	sub-01	sub-04	sub-05	sub-06	sub-07	sub-08	sub-09	sub-11	sub-12	sub-13	sub-14	sub-15
Tasks													
**MTT 1**		tSNR drop at the bottom of cerebellum					No data			tSNR drop at the bottom of cerebellum		tSNR drop at the bottom of cerebellum	
**MTT 2**		tSNR drop at the bottom of cerebellum	tSNR drop at the bottom of cerebellum		tSNR drop at the bottom of cerebellum		No data						
**Preference**			tSNR drop at the bottom of cerebellum		tSNR drop at the bottom of cerebellum	tSNR drop at the bottom of cerebellum	No data	tSNR drop at the bottom of cerebellum	tSNR drop at the bottom of cerebellum	tSNR drop at the bottom of cerebellum	tSNR drop at the bottom of cerebellum	tSNR drop at the bottom of cerebellum	Run “Food-PA” contains only the first 50 trials
									Run “Painting-AP” is missing and Run “Faces-AP” was acquired twice				
**TOM**			tSNR drop at the bottom of cerebellum				No data						
**Enumeration**			tSNR drop at the bottom of cerebellum				No data					tSNR drop at the bottom of cerebellum	tSNR drop at the bottom of cerebellum
**Self**							No data						

Four anomalies are summarized over MRI sessions and participants: tSNR drop at the bottom of the cerebellum; acquisition of two Runs “Faces-AP” in detriment of no acquisition of Run “Painting-AP” of the Preference battery for subject 11; only the first fifty trials of Run “Food-PA” of the Preference battery were registered for subject 15, since this participant could not complete the sixty trials within the pre-established duration of the run; no data for the tasks featuring this release were collected for subject 8, which will integrate a future release upon announcement.

**Online-only Table 3:** Response accuracy (%) of behavioral performance for the MTT tasks during the Event + Response conditions.

Number of trials per run = 20 / Chance level = 50%												
Task	Run id	sub-01	sub-04	sub-05	sub-06	sub-07	sub-09	sub-11	sub-12	sub-13	sub-14	sub-15
MTT WE	0	81	83	76	71	57	82	42	76	57	70	n/a
	1	81	92	78	83	60	90	51	76	58	78	95
	2	91	100	68	80	63	86	55	77	63	76	98
	**Mean**	**85**	**92**	**75**	**78**	**60**	**86**	**50**	**77**	**60**	**75**	**97**
MTT SN	0	86	93	76	73	62	83	65	71	65	73	n/a
	1	88	96	80	73	72	92	67	76	53	68	n/a
	2	91	100	56	82	68	87	70	70	57	72	n/a
	**Mean**	**89**	**97**	**71**	**77**	**68**	**88**	**68**	**72**	**59**	**72**	**n/a**
	**Total**	**87**	**94**	**73**	**78**	**64**	**87**	**59**	**75**	**59**	**73**	**97**

These scores were estimated considering the answers provided during the Event + Response conditions; the time window of the answers for a certain Event were considered from the second half of presentation of the event itself up to the first half of presentation of the next event, totalizing a period of 5 seconds. The mean of the individual scores across runs per task is also presented along with the total mean comprising both tasks. The average ± SD comprising both tasks across participants are 76 ± 13%.

*Low scores for subject 15 relate to loss of behavioral data during acquisition time in both tasks; we stress this issue is due to loss of the log files generated by the stimulus-delivery software and, thus, agnostic to subject’s performance.

**Online-only Table 4:** Response accuracy (%) of behavioral performance for the Theory-of-Mind localizer.

Number of trials per run = 10 / Chance level = 50%											
Run id	sub-01	sub-04	sub-05	sub-06	sub-07	sub-09	sub-11	sub-12	sub-13	sub-14	sub-15
0	90	90	70	70	80	n/a	90	70	90	80	90
1	80	80	80	90	60	50	70	80	70	70	70
**Mean**	**85**	**85**	**75**	**80**	**70**	**50**	**80**	**75**	**80**	**75**	**80**

The mean of the scores for the two runs is also provided for every subject. The average ± SD across participants are 74 ± 16%.

**Online-only Table 5:** Response accuracy (%) of behavioral performance per numerosity for the VSTM task.

Number of trials per run = 144 / Chance level = 50%												
Numerosity	Run id	sub-01	sub-04	sub-05	sub-06	sub-07	sub-09	sub-11	sub-12	sub-13	sub-14	sub-15
All numerosities	0	36	51	28	53	44	49	58	35	29	33	40
	1	49	49	12	57	36	56	50	40	35	18	38
	2	40	49	42	58	46	44	56	49	42	39	42
	3	43	58	39	57	39	56	57	47	43	43	44
	**Mean**	**42**	**52**	**30**	**56**	**41**	**51**	**55**	**43**	**37**	**33**	**41**
1	0	33	57	38	56	45	78	77	38	36	54	60
	1	60	59	6	60	31	60	64	55	31	18	56
	2	43	62	36	64	58	29	83	44	62	33	25
	3	70	73	54	60	50	71	58	88	62	42	50
	**Mean**	**52**	**63**	**34**	**60**	**46**	**60**	**70**	**56**	**48**	**37**	**48**
2	0	50	82	54	54	80	58	59	36	38	43	31
	1	58	38	18	55	29	75	57	40	27	40	27
	2	27	50	58	73	67	41	58	54	31	38	20
	3	31	83	17	77	58	43	67	55	45	45	56
	**Mean**	**42**	**63**	**37**	**65**	**58**	**54**	**60**	**46**	**35**	**42**	**34**
3	0	27	50	36	100	64	38	17	46	42	50	50
	1	44	64	23	60	54	55	42	82	42	12	42
	2	42	45	40	62	58	44	75	85	62	33	50
	3	58	38	71	45	33	47	50	45	27	27	36
	**Mean**	**43**	**49**	**42**	**67**	**52**	**46**	**46**	**64**	**43**	**30**	**44**
4	0	30	31	20	61	36	25	100	25	20	14	20
	1	50	88	11	50	40	67	60	31	21	20	36
	2	23	42	29	75	22	64	27	50	38	36	38
	3	9	67	40	62	27	54	56	43	45	50	45
	**Mean**	**28**	**57**	**25**	**62**	**31**	**52**	**61**	**37**	**31**	**30**	**35**
5	0	50	58	17	33	50	46	62	40	18	14	62
	1	42	8	8	50	31	36	55	7	46	0	44
	2	62	54	33	46	40	40	40	11	23	42	62
	3	45	45	33	55	33	67	64	33	36	50	36
	**Mean**	**50**	**41**	**23**	**46**	**38**	**47**	**55**	**23**	**31**	**26**	**51**
6	0	29	44	8	18	17	54	38	21	20	33	21
	1	30	38	9	62	33	36	31	40	44	20	20
	2	44	36	54	38	25	46	55	36	42	50	67
	3	47	46	9	36	33	45	46	38	33	50	47
	**Mean**	**38**	**41**	**20**	**38**	**27**	**45**	**42**	**34**	**35**	**38**	**39**

The number of bars presented in the visual stimuli was different trial-by-trial, ranging from 1 to 6. For each level of numerosity, scores in every run are related to the trials referring to visual stimuli matching the specified numerosity. The mean of the scores across runs is also provided for every subject and numerosity.

**Online-only Table 6:** Group-level scores (%) of behavioral performance per numerosity for the VSTM task.

Numerosity	Mean	SD
All numerosities	44	8
1	52	10
2	49	11
3	48	10
4	41	14
5	39	11
6	36	7

The number of bars presented in the visual stimuli ranged from 1 to 6. For each level of numerosity, the mean of the scores across subjects is provided along with its standard deviation. Although there is a successive decrease of the scores as numerosity increases, one can see a prominent step decrease for *numerosity* >3.

**Online-only Table 7:** Response accuracy (%) of behavioral performance per numerosity for the Enumeration task.

Number of trials per run = 192 / Chance level = 12.5%												
Numerosity	Run id	sub-01	sub-04	sub-05	sub-06	sub-07	sub-09	sub-11	sub-12	sub-13	sub-14	sub-15
All numerosities	0	72	67	49	58	69	69	58	69	65	34	54
	1	70	71	47	69	73	69	51	66	64	48	50
	**Mean**	**71**	**69**	**48**	**64**	**71**	**69**	**54**	**68**	**64**	**41**	**52**
1	0	100	77	93	89	100	93	100	86	100	82	75
	1	92	100	60	100	92	70	100	100	100	82	83
	**Mean**	**96**	**88**	**76**	**94**	**96**	**82**	**100**	**93**	**100**	**82**	**79**
2	0	100	93	83	80	100	73	92	85	90	80	88
	1	100	85	92	100	94	78	94	100	100	56	100
	**Mean**	**100**	**89**	**88**	**90**	**97**	**76**	**93**	**92**	**95**	**68**	**94**
3	0	100	78	100	69	83	100	69	92	87	25	77
	1	100	100	64	73	100	100	42	91	89	80	64
	**Mean**	**100**	**89**	**82**	**71**	**92**	**100**	**56**	**92**	**88**	**52**	70
4	0	100	87	33	47	62	90	0	92	89	18	40
	1	92	89	33	67	88	79	0	83	100	36	64
	**Mean**	**96**	**88**	**33**	**57**	**75**	**84**	**0**	**88**	**94**	**27**	**52**
5	0	17	27	18	23	58	64	45	36	0	18	14
	1	42	47	31	45	58	80	20	38	17	33	30
	**Mean**	**30**	**37**	**24**	**34**	**58**	**72**	**32**	**37**	**8**	**26**	**22**
6	0	18	31	8	29	54	18	60	45	44	0	11
	1	23	62	17	20	64	38	44	62	20	27	33
	**Mean**	**20**	**46**	**12**	**24**	**59**	**28**	**52**	**54**	**32**	**14**	**22**
7	0	50	58	13	80	71	55	55	50	69	12	36
	1	36	60	22	89	60	69	67	36	73	36	8
	**Mean**	**43**	**59**	**18**	**84**	**66**	**62**	**61**	**43**	**71**	**24**	**22**
8	0	67	78	50	71	30	44	38	50	36	50	73
	1	93	56	50	47	29	47	33	33	10	47	38
	**Mean**	**80**	**67**	**50**	**59**	**30**	**46**	**36**	**42**	**23**	**48**	**56**

The number of bars presented in the visual stimuli ranged from 1 to 8. For each level of numerosity, scores in every run are related to the trials referring to visual stimuli matching the specified numerosity. The mean of the scores across runs is also provided for every subject and numerosity.

**Online-only Table 8:** Group-level scores (%) of behavioral performance per numerosity for the Enumeration task.

Numerosity	Mean	SD
All numerosities	61	10
1	90	8
2	89	9
3	81	16
4	63	30
5	35	17
6	33	16
7	50	21
8	49	16

The number of bars presented in the visual stimuli ranged from 1 to 8. For each level of numerosity, the mean of the scores across subjects is provided along with its standard deviation. Although there is a successive decrease of the scores as numerosity increases, one can see a prominent step decrease for *numerosity* >4.

**Online-only Table 9:** Response accuracy (%) of behavioral performance for the recognition phase of the Self task.

Chance level = 50%												
Run id	No. of trials^*^	sub-01	sub-04	sub-05	sub-06	sub-07	sub-09	sub-11	sub-12	sub-13	sub-14	sub-15
0	72	100	91	47	91	91	94	87	86	81	87	90
1	72	90	84	50	84	91	90	88	90	72	73	84
2	72	94	87	81	90	90	86	81	84	76	80	83
3	96	2	90	88	79	93	98	82	82	76	81	94
**Mean**	**—**	**72**	**89**	**67**	**86**	**92**	**92**	**85**	**86**	**77**	**81**	**88**

The mean of the scores for the four runs is also provided for every subject. The average ± SD across participants are 83 ± 8%.

*“No. of trials” refer (in this table) to the number of trials only for the recognition phase in the specified run and, thus, not to the total number of trials in the run. Because run #3 was longer than the remainder ones, the number of trials for the recognition phase was therefore greater. Concretely, this run included four blocks for the recognition phase with twenty four trials each, whereas all others comprised three blocks.

**Online-only Table 10:** Cognitive labels associated with the model features, involved in the main contrasts, implemented for each task of the second-dataset release.

Task	Description of the Condition^§^	Cognitive Labels
MTT battery^*^	all references in we/sn island	auditory perception
		memory retrieval
		spatial localization
		cardinal-direction judgment
		temporal cognition
	space cue on all references in we/sn island	auditory perception
		semantic categorization
		cardinal orientation
	time cue on all references in we/sn island	auditory perception
		semantic categorization
		time orientation
	westside/southside events	auditory perception
		spatial localization
		west/south cardinal-direction judgment
	eastside/northside events	auditory perception
		spatial localization
		east/north cardinal-direction judgment
	we/sn past events	auditory perception
		temporal cognition
		past time
	we/sn future events	auditory perception
		temporal cognition
		future time
	responses to events in we/sn island	response selection
		response execution
Preference Food	food evaluation	food cue reactivity
		judgment
	food preference	food cue reactivity
		reward valuation
		incentive salience
	confidence in food preference	food cue reactivity
		confidence judgment
Preference Painting	painting evaluation	visual form discrimination
		visual color discrimination
		judgment
	painting preference	visual form discrimination
		visual color discrimination
		reward valuation
		incentive salience
	confidence in painting preference	visual form discrimination
		visual color discrimination
		confidence judgment
Preference Face	face evaluation	visual face recognition
		face perception
		facial attractiveness recognition
		judgment
	face preference	visual face recognition
		face perception
		facial attractiveness recognition
		reward valuation
		incentive salience
	confidence in face preference	visual face recognition
		face perception
		facial attractiveness recognition
		confidence judgment
Preference House	house evaluation	visual place recognition
		judgment
	house preference	visual place recognition
		reward valuation
		incentive salience
	confidence in house preference	visual place recognition
		confidence judgment
TOM localizer	fact judgments	semantic processing
		reading
		response execution
		response selection
		reasoning
		theory-of-mind
	belief judgments	semantic processing
		reading
		response execution
		response selection
		reasoning
EP localizer	emotional-pain story	reading
		response execution
		response selection
		empathy
		imagined emotional pain
		theory-of-mind
	physical-pain story	reading
		response execution
		response selection
		empathy
		imagined physical pain
MOV localizer	mental movie	visual attention
		empathy
		imagined emotional pain
		theory-of-mind
	physical-pain movie	visual attention
		empathy
		imagined physical pain
VSTM	vstm response to constant numerosity	visual orientation
		visual form discrimination
		shape recognition
		short-term memory
		response selection
		response execution
	vstm response to numerosity	visual working memory
		numerosity
	vstm response to numerosity interaction	visual buffer
		numerosity
Enumeration	enumeration response to constant numerosity	enumeration
		numerosity
		shape recognition
		response selection
		response execution
	enumeration response to numerosity	visual working memory
		numerosity
	enumeration response to numerosity interaction	visual buffer
		numerosity
Self	instructions	reading
	self-reference encoding	judgment
		reading
		response selection
		response execution
		self-reference effect
	other-reference encoding	judgment
		reading
		response selection
		response execution
	self-reference recognition	recognition
		reading
		response selection
		response execution
		self-reference effect
	other-reference recognition	recognition
		reading
		response selection
		response execution
		other-reference effect
	correct rejection	memory
		reading
		response selection
		response execution
	no recognition	response selection
		response execution
Bang	speech	speech perception
		speech processing
		language comprehension
		language processing
		action preception
		audiovisual perception
		auditory scene analysis
		social cognition
	no speech	action perception
		audiovisual perception
		auditory scene analysis
		social cognition

Tags were obtained from the Cognitive Atlas: https://www.cognitiveatlas.org. These labels provide an approximate description of the underlying cognitive components that are involved in the performance of the tasks.

*“MTT battery” comprises both MTT WE and MTT SN tasks. Conditions and their cognitive labels were defined in the same way for both of them.
